# Identification of genes regulated by histone acetylation during root development in *Populus trichocarpa*

**DOI:** 10.1186/s12864-016-2407-x

**Published:** 2016-02-04

**Authors:** Xujun Ma, Chao Zhang, Bing Zhang, Chuanping Yang, Shujuan Li

**Affiliations:** State Key Laboratory of Tree Genetics and Breeding (Northeast Forestry University), Northeast Forestry University, Harbin, 150040 China

**Keywords:** *Populus trichocarpa*, Histone deacetylase, Trichostatin A, Digital gene expression

## Abstract

**Background:**

Histone deacetylases (HDACs) are key enzymes catalyzing the removal of acetyl groups from histones. HDACs act in concert with histone acetyltransferases (HATs) to regulate histone acetylation status, which modifies chromatin structure, affecting gene transcription and thus regulating multiple biological processes such as plant growth and development. Over a decade, certain HDACs in herbaceous plants have been deeply studied. However, functions of HDACs in woody plants are not well understood.

**Results:**

Histone deacetylase specific inhibitor trichostatin A (TSA) was used to investigate the role of HDACs in organogenesis of roots and root development in *Populus trochocarpa*. The adventitious roots were regenerated and grown on medium supplemented with 0, 1, and 2.5 μM TSA. TSA treatment delayed root regeneration and inhibited primary root growth. To examine the genes modified by TSA in the regenerated roots, tag-based digital gene expression (DGE) analysis was performed using Illumina HiSeqTM 2000. Approximately 4.5 million total clean tags were mapped per library. The distinct clean tags for the three libraries corresponding to 0, 1 and 2.5 μM TSA treatment were 166167, 143103 and 153507, from which 38.45 %, 31.84 % and 38.88 % were mapped unambiguously to the unigene database, respectively. Most of the tags were expressed at similar levels, showing a < 5-fold difference after 1 μM and 2.5 μM TSA treatments and the maximum fold-change of the tag copy number was around 20. The expression levels of many genes in roots were significantly altered by TSA. A total of 36 genes were up-regulated and 1368 genes were down-regulated after 1 μM TSA treatment, while 166 genes were up-regulated and 397 genes were down-regulated after 2.5 μM TSA treatment. Gene ontology (GO) and pathway analyses indicated that the differentially expressed genes were related to many kinds of molecular functions and biological processes. The genes encoding key enzymes catalyzing gibberellin biosynthesis were significantly down-regulated in the roots exposed to 2.5 μM TSA and their expression changes were validated by using real-time PCR.

**Conclusions:**

HDACs were required for *de novo* organogenesis and normal growth of populus roots. DGE data provides the gene profiles in roots probably regulated by histone acetylation during root growth and development, which will lead to a better understanding of the mechanism controlling root development.

**Electronic supplementary material:**

The online version of this article (doi:10.1186/s12864-016-2407-x) contains supplementary material, which is available to authorized users.

## Background

Histone acetylation, as a major and important post-translational modification of core histones, was started to be investigated early in 1964 [1]. Histone acetylation modifies chromatin structure, affecting gene transcription and thus regulating multiple cellular processes. Histone acetylation and deacetylation were regulated by histone acetyltransferases (HATs) and histone deacetylases (HDACs), respectively. HATs add acetyl groups to lysines on core histones, while HDACs remove the acetyls from histones. Histone acetylation catalyzed by HATs leads to the expanded structure of chromatin, while hypoacetylation of histones mediated by HDACs is generally associated with the condensed structure of chromatin and repression/silencing of genes [2]. HDACs are widely distributed in eukaryotes, including animals, plants and fungi. To date, HDACs in human and animals have been more widely and deeply investigated than plants. In 1988, HDAC enzyme activity was first detected in pea [3]. However, only in recent years, HDACs in plants have attracted more attention and certain HDAC genes in Arabidopsis and crops have been deeply studied [4]. The available data from these plants showed that HDACs played a key role in plant growth, development and stress responses [4]. Based on sequence homology to yeast HDACs, HDACs in plants were divided into three major groups, namely reduced potassium dependency 3/histone deacetylase 1 (RPD3/HDA1), histone deacetylase 2 (HD2) and silent information regulator 2 (SIR2). The enzyme activity of RPD3/HDA1- and HD2-type histone deacetylases could be inhibited by HDAC specific inhibitors trichostatin A (TSA) and butyrate (NaB) [2]. The genome of Black cottonwood (Populus trichocarpa Torr. & Gray) was sequenced in 2006 [5] and eleven HDAC genes were identified in the Populus genus. However, functions of these HDACs are remaining to be characterized.

In plants, root system development such as root hair development, lateral root formation and primary root growth were epigenetically regulated by HDACs. Early in 2000, Murphy et al. found that TSA and helminthosporium carbonum (HC) toxin were able to halt mitosis in cultured root meristems of *Pisum sativum* [6]. Recently, HDACs have been reported to be involved in root hair development. For example, in Arabidopsis, TSA treatment altered the cellular pattern of root epidermis and induced hair cell development at non-hair positions [7]. Moreover, HDA18 was further identified to be a key regulator of root development. Ethylene insensitive 3 (EIN3) and its closest homolog ein3-like1 (EIL1) are two transcription factors that integrate ethylene signaling and jasmonic acid (JA) signaling in root development. Arabidopsis RPD3-type histone deacetylase HDA6 was able to repress EIN3/EIL1-dependent transcription and inhibit JA signaling [8]. When the Arabidopsis seedlings were subjected to TSA, root hair formation was drastically induced. HDACs also play an important role in lateral root (LR) development. LR formation is a progress regulated by auxin and auxin response factors (ARFs), such as ARF7 and ARF19. In Arabidopsis, *arf7/arf19* double mutants had few LRs [9, 10]. IAA14 is an indole-3-acetic acid (IAA) regulatory protein and functions as a repressor of ARF proteins in Arabidopsis. Its gain-of-function mutation in domain II, *slr-1*, improved its stability and resulted in constitutive inactivation of ARF functions [11]. As a result, auxin-induced pericycle cell divisions for LR initiation were blocked. However, the LR formation in *slr-1* mutant plants was promoted by TSA treatment [11]. Thus HDACs appeared to be required for the LR phenotype in *slr-1* mutants and play a negative role in the activation of ARF7/19 functions in LR initiation. In addition, HDACs acted as an important regulator in primary root growth. The distribution of auxin in primary root tips was mediated by auxin transporters such as pin-formed (PIN) family and ATP-binding cassette (ABC) superfamily [12]. In the work of Nguyen et al., the primary root elongation was significantly inhibited by HDAC inhibitors TSA and NaB [13]. In response to HDAC inhibitors, the accumulation of Arabidopsis PIN1 protein in root tips was abolished through the 26S proteasome-mediated degradation [13]. In rice, overexpression of *OsHDAC1* gene in transgenic seedlings enhanced root growth [14] and a NAM-ATAF-CUC (NAC) transcription factor OsNAC6, which mediates the alteration of root development, was identified to be the target of OsHDAC1 [15]. These findings indicated that HDACs act as key regulators in root system development.

The Black cottonwood (Populus trichocarpa Torr. & Gray) is of considerable commercial and ecological importance and accepted as a model system for biological study of trees [16]. In the Populus genus, an increasing number of genes involved in development and stress responses have been identified at genome level. For example, genes encoding heat shock proteins (Hsps) and heat shock factors (Hsfs) in response to drought stress have recently been identified and comprehensively characterized [17–19]. Meanwhile, using high-throughput RNA-Seq method, long intergenic non-coding RNAs (lncRNAs) [20], drought responsive microRNAs [21] and alternative splicing of xylem-expressed genes [22] were genome-widely identified. Thus the high-throughput RNA sequence analysis is an effective method to identify genes involved in development and stress responses in the Populus.

For woody plants, *de novo* organogenesis under tissue culture conditions is an effective method to reproduce seedlings. However, for many woody plants, shoot or root regeneration was still difficult, even after trying many methods such as changing hormones, medium components, or culture conditions. To date, the role of epigenetic regulation on organogenesis was less known. Investigation of HDAC functions in root organogenesis and development and identification of genes regulated by HDACs at genome-wide level will provide valuable information for understanding root development mechanism. In this study, histone deacetylase specific inhibitor TSA was used to investigate the role of HDACs in populus root regeneration and development. Our results showed that TSA treatment decreased HDAC activity in roots, delayed root regeneration and inhibited primary root growth. A digital gene expression (DGE) analysis was performed to examine the differentially expressed genes in roots when subjected to different concentrations of TSA. Our findings suggested that root organogenesis and development were epigenetically regulated in *Populus trichocarpa*.

## Results

### TSA modified root regeneration and root system development

**Fig. 1:**
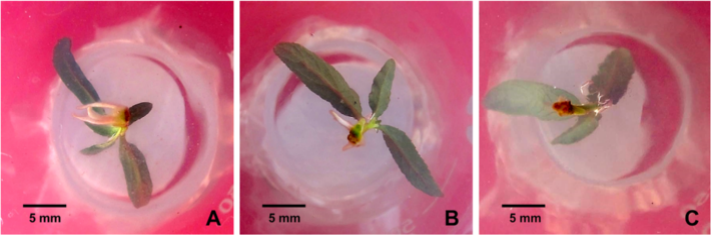
Inhibition of root regeneration by TSA. Roots were regenerated from the bottom of shoots which were cultured on medium supplemented with 0 μM (**a**), 1 μM (**b**) and 2.5 μM (**c**) TSA in *Populus trichocarpa*

**Fig. 2:**
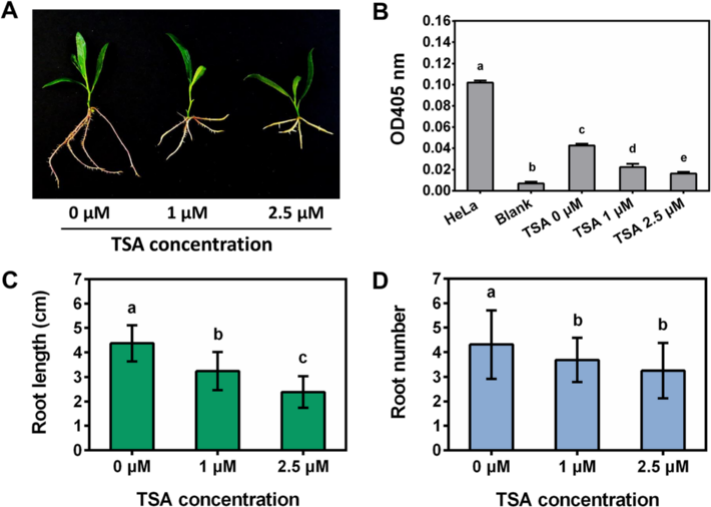
Inhibition of root growth by TSA. The growth of regenerated roots was inhibited when subjected to indicated concentrations of TSA for 2 weeks (**a**). TSA inhibited HDAC activities in the regenerated roots (**b**). The length of regenerated roots (**c**) and root number (**d**) were decreased by TSA in a dose-dependent manner

**Fig. 3:**
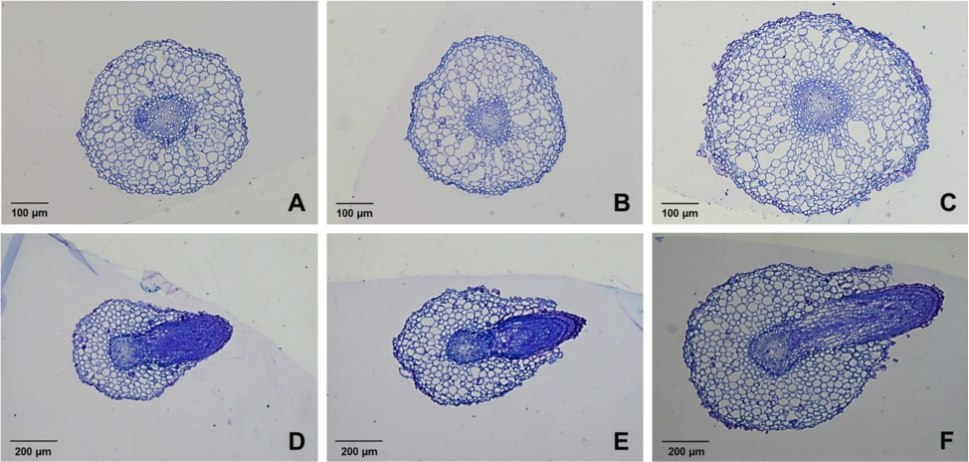
Morphology of roots after growth on medium supplemented with 0, 1 and 2.5 μM TSA, respectively. **a**-**c**, cross sections of root tips; **d**-**f**, cross sections in the middle region of the roots. **a** and **d**, roots after growth on medium without TSA supplemented for two weeks; **b** and **e**, roots after growth on medium containing 1 μM TSA; **c** and **f**, roots after growth on medium containing 2.5 μM TSA

The populus shoots were transferred onto the rooting medium (woody plant medium, WPM) supplemented with 0, 1 and 2.5 μM TSA to examine the role of TSA on root regeneration, growth and development. At each concentration, at least 45 shoots were cultured on the medium for root regeneration and the regenerated roots showed the same morphological traits. After shoots being transferred onto the rooting medium without TSA supplemented for 6 d, roots were regenerated from the bottom of shoots and reached around 1 cm in length, while in presence of TSA, the regeneration of roots was delayed. On the medium containing 1 μM TSA, the length of the regenerated roots was about 0.5 cm, while no root was regenerated on the medium containing 2.5 μM TSA (Fig. 1). The growth of the regenerated roots was inhibited by TSA after shoots being transferred onto the rooting medium for 2 weeks (Fig. 2a). HDAC activities in the regenerated roots were 1.9-fold and 2.6-fold decreased after 1 μM and 2.5 μM TSA treatment, respectively (Fig. 2b). The length (Fig. 2c) and number (Fig. 2d) of the regenerated roots were significantly reduced by TSA in a dose-dependent manner. In addition, the regenerated roots growing on the medium containing 2.5 μM TSA were much thicker than control roots. To know the reason why the roots were so thick, analysis of semithin sections was performed. The morphological analysis showed that the number of cells in cortex was increased, while the size of the cells appeared not to be significantly altered (Fig. 3). These findings suggested that HDACs were required for root organogenesis, growth and development in populus.

### Digital gene expression (DGE) libraries and tag mapping

**Fig. 4:**
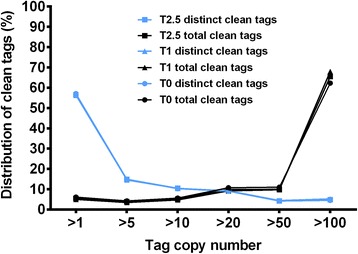
Distribution of clean tags in three libraries. Black color represents the distribution of total clean tags and blue color represents the distribution of distinct clean tags in T0, T1 and T2.5 libraries, corresponding to 0 μM (T0), 1 μM (T1), and 2.5 μM TSA treatment (T2.5), respectively

In order to know the possible mechanism by which root growth and development were regulated by TSA, a DGEs analysis was performed. The DGE libraries from the roots grown on the WPM medium supplemented with 0, 1, and 2.5 μM TSA were named libraries T0, T1 and T2.5, respectively (Table 1). A total of 4816584, 4906668 and 4805265 raw tags were sequenced in T0, T1 and T2.5 libraries, respectively. After filtering out the adaptors, low quality tags containing unknown nucleotides “N” and tags with copy number < 2, the remaining “clean” tags for the three libraries were 4453843 (92.4 %), 4620879 (94.2 %) and 4504396 (93.7 %), respectively. The distinct tags for the three libraries corresponding to 0, 1 and 2.5 μM TSA treatment were 372060, 322043 and 338137, from which the distinct clean tags were 166167 (44.7 %), 143103 (44.4 %) and 153507 (45.3 %), respectively. The distribution of the total and distinct clean tag copy numbers showed highly similar tendencies in the three libraries (Fig. 4). The percentage of distinct clean tags declined with the increase of tag copy number (Fig. 4). For each library, approximately 71 % of the transcripts were expressed at low levels (<10 copies), less than 24 % of the distinct clean tags had copy numbers between 11 and 100, while only ~5 % were expressed at high levels (>100 copies). Therefore, most of the genes in roots were expressed at low levels and only a small number of the genes were expressed at high levels.

**Table 1:** Statistics of categorization and abundance of DGE tags

Summary		T0	T1	T2.5
Raw Data	Total	4816584	4906668	4805265
	Distinct Tag	372060	322043	338137
Clean Tag	Total number	4453843	4620879	4504396
	Distinct Tag number	166167	143103	153507
All Tag Mapping to Gene	Total number	1211139	889173	1180631
	Total % of clean tag	27.19 %	19.24 %	26.21 %
	Distinct Tag number	64057	45678	59848
	Distinct Tag % of clean tag	38.55 %	31.92 %	38.99 %
Unambiguous Tag Mapping to Gene	Total number	1207720	885861	1176439
	Total % of clean tag	27.12 %	19.17 %	26.12 %
	Distinct Tag number	63895	45557	59683
	Distinct Tag % of clean tag	38.45 %	31.84 %	38.88 %
All Tag-mapped Genes	number	20423	17990	19966
	% of ref genes	45.35 %	39.95 %	44.34 %
Unambiguous Tag-mapped Genes	number	20376	17947	19907
	% of ref genes	45.25 %	39.85 %	44.21 %
Mapping to Genome	Total number	2909567	3341569	3038306
	Total % of clean tag	65.33 %	72.31 %	67.45 %
	Distinct Tag number	83956	78922	78929
	Distinct Tag % of clean tag	50.53 %	55.15 %	51.42 %
Unknown Tag	Total number	333137	390137	285459
	Total % of clean tag	7.48 %	8.44 %	6.34 %
	Distinct Tag number	18154	18503	14730
	Distinct Tag % of clean tag	10.93 %	12.93 %	9.60 %

To reveal the molecular events behind DGE profiles, clean tags of the three DGE libraries were mapped to the *Populus trichocarpa* genome, allowing only a 1-bp mismatch (Table 1). For T0, T1 and T2.5 DGE libraries, 50.53 %, 55.15 % and 51.42 % of the distinct clean tags were mapped to the populus genome database, 38.45 %, 31.84 % and 38.88 % of the distinct clean tags were mapped unambiguously to the unigene database, and 10.93 %, 12.93 % and 9.6 % of the distinct clean tags did not map to the unigene virtual tag database, respectively.

### Differentially expressed genes

**Fig. 5:**
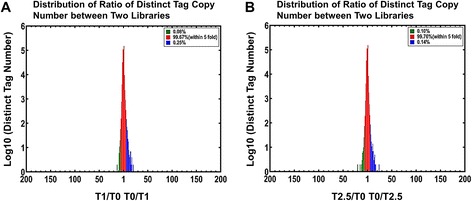
Differentially expressed tags in two libraries. The tags were expressed at similar levels in T1 (**a**) and T2.5 (**b**) libraries. The “x” axis represents fold-change of differentially expressed distinct tags in two libraries. The “y” axis represents the number of distinct tags. The region in red color indicates distinct tags with a <5-fold fold-change in T1 and T2.5 libraries. The regions in green and blue indicate the distinct tags up- and down-regulated for more than 5-folds in T1 and T2.5 libraries, respectively

**Fig. 6:**
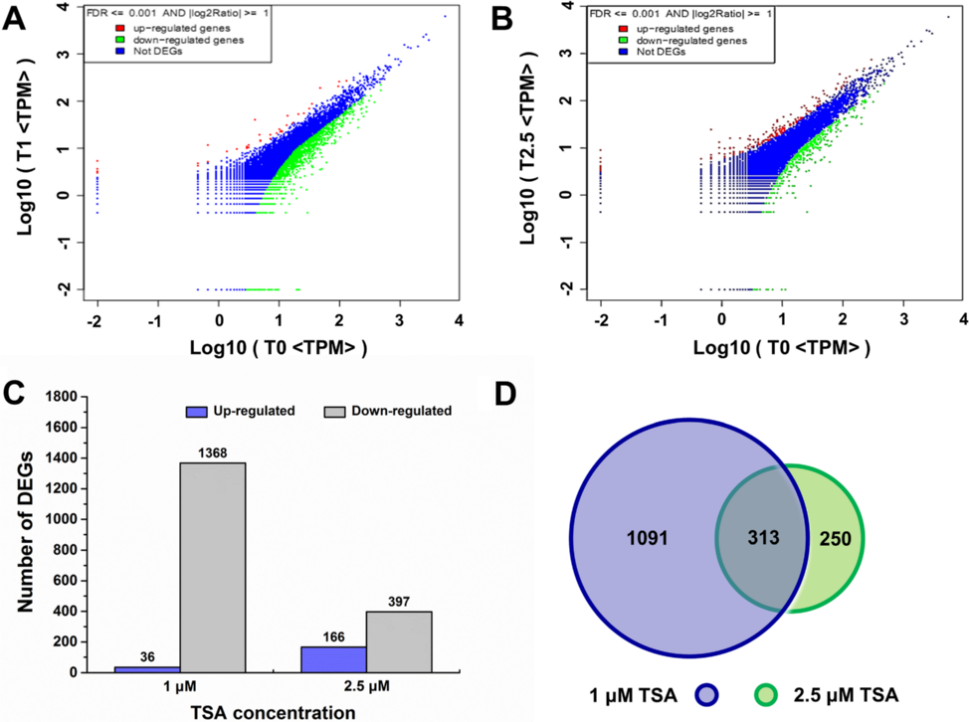
Differential expression analysis of unigenes by DGE. The expression level for each unigene in roots after growth on medium supplemented with TSA is shown in the volcano plots (**a**) and (**b**). The “x” axis represents the log10 of transcripts per million of the control (0 μM TSA treatment) and the “y” axis represents the log10 of transcripts per million of samples treated by 1 μM TSA (**a**) and 2.5 μM TSA (**b**). The red dots indicate the abundance of transcripts higher than two folds and green dots indicate transcripts lower than two folds in T1 and T2.5 libraries in comparison with T0 library. FDR ≤ 0.001 and the absolute value of log2Ratio ≥ 1 were used as the threshold to judge the significance of gene expression difference. The number of up-regulated and down-regulated unigenes in roots after TSA treatments (**c**) and the common differentially expressed genes after 1 μM TSA and 2.5 μM TSA treatments (**d**) were summarized

To identify the differentially expressed genes (DEGs) in T0, T1 and T2.5 libraries, a rigorous algorithm was developed. The tag frequencies that appeared in libraries were used for estimating gene expression levels. The distribution of fold-changes of tag copy number showed that most of the tags were expressed at similar levels, showing a < 5-fold difference after 1 μM and 2.5 μM TSA treatments and the maximum fold-change of the tag copy number was around 20 (Fig. 5). After 1 μM TSA treatment, the expression levels of 99.67 % tags were < 5-fold changed, while only 0.08 % tags were up-regulated by at least five folds and 0.25 % tags were down-regulated by at least five folds (Fig. 5a). After 2.5 μM TSA treatment, the expression levels of 99. 76 % tags were < 5-fold changed, while only 0.1 % tags were up-regulated by at least five folds and 0.14 % tags were down-regulated by at least five folds (Fig. 5b). In our study, a threshold of false discovery rate (FDR) ≤ 0.001 and 2- fold change in expression level were used to judge the significance of gene expression difference (Fig. 6a and b). The red dots represented the abundance of transcripts higher than two folds and green dots represented transcripts lower than two folds in T1 and T2.5 libraries in comparison with T0 library. The blue dots represented the abundance of transcripts less than two folds. A total of 1404 genes and 563 genes were detected to be differentially expressed in T1 and T2.5 libraries in comparison with T0 data set, respectively (Fig. 6c). Of the differentially expressed genes, 313 genes were present in both T1 and T2.5 libraries (Fig. 6d). After TSA treatment, most of the genes in roots were down-regulated. Among all differentially expressed genes, 36 were up-regulated and 1368 were down-regulated after 1 μM TSA treatment, while 166 were up-regulated and 397 were down-regulated after 2.5 μM TSA treatment (Fig. 6c). Most of the differentially expressed genes had functional annotations, while some of the genes were not well characterized and of unknown functions.

### Gene ontology functional analysis of DEGs

**Fig. 7:**
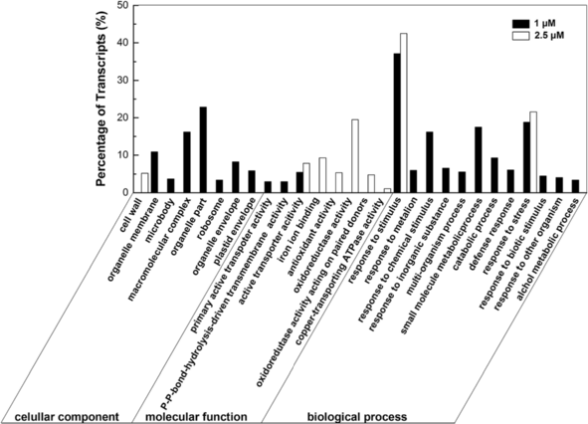
Histogram showing Gene Ontology functional enrichment analysis of DEGs. Transcripts were classified into three different categories, cellular component, molecular function and biological process

To better understand the biological functions of the differentially expressed genes (DEGs), gene ontology (GO) enrichment analysis was performed. GO includes three ontologies, cellular component, molecular function and biological process, describing properties of genes and their products in any organism. Each of the ontologies is composed of GO-terms. GO enrichment analysis applies a hypergeometric test to map all DEGs to terms in the GO database, searching for significantly enriched GO terms which are defined by a threshold of corrected-P value ≤ 0.05. A total of 1182 DEGs in T1 library and 448 DEGs in T2.5 in comparison with T0 library were classified into the three main ontologies, respectively. For T1 library in comparison with T0, 616 genes were mapped to the significantly enriched GO terms (P ≤ 0.05) and could be categorized into 22 functional groups (Fig. 7). For T2.5 library in comparison with T0, 208 genes were mapped to the significantly enriched GO terms (P ≤ 0.05) and could be categorized into 9 functional groups (Fig. 7). In the presence of 1 μM TSA, most of the differentially expressed genes were associated with cellular component or involved in biological processes. However, under 2.5 μM TSA treatment, most of the differentially expressed genes were specially categorized into the ontology of molecular function, including GO terms “active transporter activity”, “iron ion binding”, “antioxidant activity”, “oxidoreductase activity”, “oxidoreductase activity acting on paired donors” and “copper-transporting ATPase activity”. For T1 and T2.5 libraries, the common GO terms include “active transporter activity”, “response to stimulus” and “response to stress”.

### Pathway analysis for DEGs

**Fig. 8:**
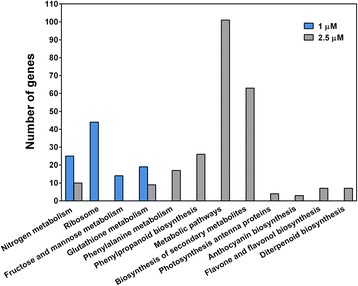
Histogram illustrating pathway enrichment analyses

To further characterize the function of the differentially expressed genes in T1 and T2.5 libraries, we mapped the genes to terms in kyoto encyclopedia of genes and genomes (KEGG) database to identify significantly enriched metabolic or signal transduction pathways (Fig. 8). Among the mapped pathways, four pathways were significantly enriched (Q value ≤ 0.05) in roots grown on the medium containing 1 μM TSA. Most of the genes in the four enriched pathways, including nitrogen metabolism (25 members) (Additional file 1), ribosome (44 members) (Additional file 2), fructose and mannose metabolism (12 out of 14 members) (Additional file 3), and glutathione metabolism (18 out of 19 members) (Additional file 4), were down-regulated. Nitrogen metabolism is important for plant growth and development. Proteins are synthesized by ribosomes. Thus the down-regulation of the genes involved in nitrogen metabolism and ribosomal protein synthesis may impair normal growth and development of roots. For the roots grown on the medium supplemented with 2.5 μM TSA, ten pathways were significantly enriched (Q value ≤ 0.05) and the top two abundant pathways were metabolic (101 members) and biosynthesis of secondary metabolites (63 members). Most of the genes in the ten pathways were down-regulated. The genes in the nitrogen metabolism pathway (10 members), photosynthesis-antenna proteins (4 members), anthocyanin biosynthesis (3 members), diterpenoid biosynthesis (6 out of 7 members), phenylpropanoid biosynthesis pathway (17 out of 26), and flavones and flavonol biosynthesis (5 out of 7 members) were down-regulated. Some genes in phenylalanine metabolism (9 out of 17 members) and glutathione metabolism (6 out of 9) were up-regulated. The genes in the metabolic pathway, diterpenoid/GA, and nitrogen metabolic may play a significant role in plant growth and development. The flavones and flavonol biosynthesis, anthocyanin biosynthesis, and phenypropanoid biosynthesis were important for plant stress tolerance [23, 24].

### Effect of TSA on the GA biosynthesis pathway

**Fig. 9:**
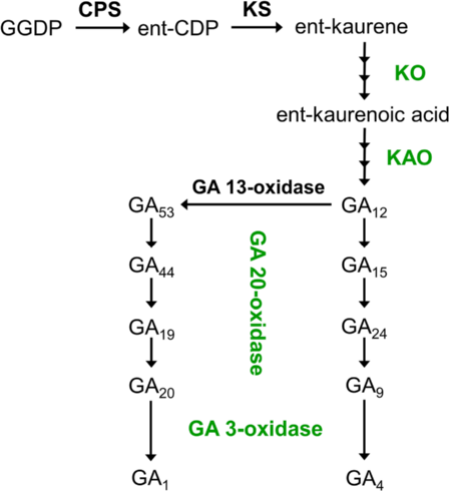
Gibberellin biosynthesis pathway in populus roots in response to 2.5 μM TSA treatment. The expression of four genes encoding enzymes catalyzing GA biosynthesis (marked with green) was down-regulated on exposure to 2.5 μM TSA for 2 weeks

**Fig. 10:**
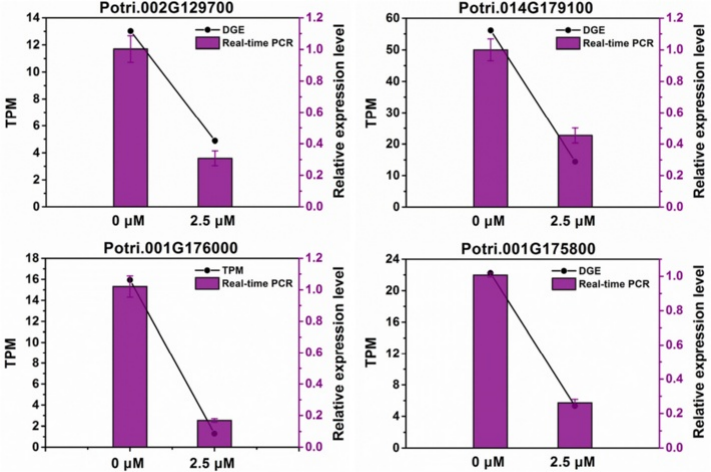
Real-time PCR validations of GA biosynthesis genes. The expression levels of genes participating in GA biosynthesis, Potri.002G129700 (*KO*), Potri.014G179100 (*KAO*), Potri.001G176000 and Potri.001G175800 (*GA20ox* and *GA3ox*), were analyzed by real-time PCR. The PCR results were consistent with the data in DGE analysis

Gibberellins (GAs), as a large family of tetracyclic diterpenoid, regulate plant growth and development such as seed germination, stem elongation, flowering, fruit development, and circadian and light regulation [25]. The growth and development of root system were regulated by GA as well. In our study, diterpenoid/GA biosynthesis pathway was significantly enriched after roots were grown on the medium supplemented with 2.5 μM TSA for two weeks. A total of four genes participating in GA biosynthesis, including *KO*, *KAO*, *GA20ox* and *GA3ox*, were detected in T2.5 library and all of them were down-regulated (Fig. 9). *KO* (Potri.002G129700) encoded enzyme ent-kaurene oxidase and *KAO* (Potri.014G179100) encoded ent-kaurenoic acid hydroxylase. Genes Potri.001G176000 and Potri.001G175800 were homologues of Arabidopsis *GA20ox* and *GA3ox* which encoded GA 20-oxidase and GA 3-oxidase. The expression levels of these genes were confirmed by real-time PCR (Fig. 10). The real-time PCR results were consistent with the data obtained by DGE analysis. These findings revealed that inhibition of HDAC enzyme activity by TSA resulted in the down-regulation of genes in GA biosynthesis pathway.

### Confirmation of differentially expressed genes by quantitative real-time PCR

**Fig. 11:**
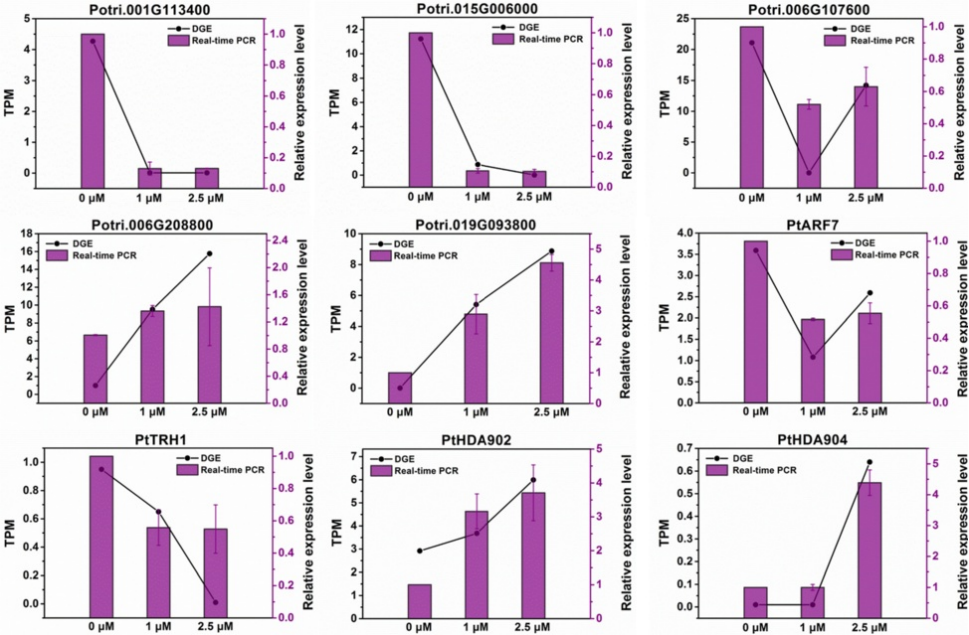
Real-time PCR validations of tag-mapped genes. Relative level, 2^−△△CT^; TPM, transcript per million mapped reads

To verify the DGE data, nine genes were selected for quantitative real-time PCR analysis (Fig. 11). The representative genes selected for the analysis included two genes related to root development, Potri.006G138500 (*auxin response factor 7*, *ARF7*)) and Potri.003G133900 (*tiny root hair 1*, *TRH1*)), and two populus HDAC genes, Potri.009G170700 (*HDA902*) and Potri.001G460000 (*HDA904*). The expression changes of the nine genes in DGE analysis were consistent with the results obtained by real-time PCR. It is interesting that populus *HDA902* and *HDA904* genes were able to be regulated by TSA.

## Discussion

### TSA modified the *de novo* organogenesis of roots

Tissue culture is an effective and fast method to obtain seedlings for many woody plants. In woody plants such as populus and birch, detached shoots are generally cultured on medium containing appropriate plant hormones and nutrient to regenerate roots. In order to improve the regeneration rate of roots, the hormones such as auxin and cytokinin, components of medium, or culture conditions such as temperature, humanity and lightening were usually adjusted. Nevertheless, for many woody plants, regeneration of adventitious roots from detached tissues or organs is still difficult. To date, it has not been fully understood that root regeneration may be epigenetically regulated. In our work, we examined the role of histone deacetylases in *de novo* root organogenesis using HDAC specific inhibitor TSA. TSA treatment inhibited populus root organogenesis in a dose-dependent manner (Fig. 1), suggesting that HDACs were required for populus root regeneration from detached shoots under tissue culture conditions. This finding might shed light on the organogenesis of those woody plants which are difficult to obtain regenerated seedlings by tissue culture method.

### Differentially expressed genes (DEGs)

Histone deacetylases (HDACs) catalyze histone deacetylation and are usually associated with the repression of gene transcription. TSA, as a HDAC specific inhibitor, is expected to have a role to increase histone acetylation levels, thus leading to up-regulation of genes. However, interestingly, most of the differentially expressed genes in roots were down-regulated on exposure to TSA, especially when roots were exposed to 1 μM TSA. It appeared that histone acetylation may have a positive or negative role in gene activation. In yeast, histone deacetylation not only repress genes but can be required for gene activation [26]. Using spotted oligo-gene microarray, Tian et al. investigated the expression of genes in *AtHDA19* T-DNA insertion mutant (*athd1-t1*) [27]. Over 7 % of the transcriptome was modified. In leaves and flowers of the *athd1-t1* mutant, relatively equal numbers of genes were up- or downregulated. These findings indicate that histone acetylation may activate or repress the transcription of genes, which is consistent with our result. In our study, the populus roots were regenerated on WPM medium containing different concentrations of TSA (0, 1 and 2.5 μM). Organogenesis and development of the roots were inhibited by TSA in a dose-dependent manner, which were consistent with the finding in Arabidopsis [28]. In order to know whether the genes were modified by TSA in a dose-dependent manner, the expression levels of DEGs in the libraries were compared. After comparison, only three genes exhibited dose-dependent manner when roots were subjected to different concentrations of TSA, suggesting that the expression of genes in response to TSA was not in a dose-dependent manner. In T1 library, most of the DEGs (1091) were not found in T2.5 library, while almost half of the DEGs in T2.5 library were not included in T1 library, indicating that different sets of genes in roots were modified at each concentration (1 and 2.5 μM TSA). The morphological difference of the roots under 1 and 2.5 μM TSA treatments might be due to different genes were modified during root development.

### Stress-responsive genes

**Fig. 12:**
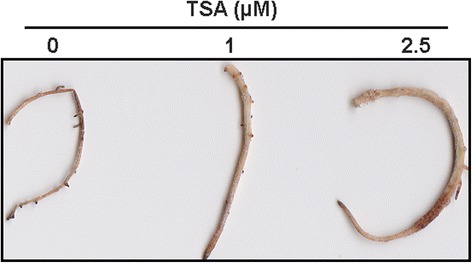
ROS accumulation in roots in response to TSA treatments. The detached shoots were transferred onto medium supplemented with indicated concentrations of TSA. After 2 weeks the regenerated roots were collected for DAB staining to check the accumulation level of H_2_O_2_

**Fig. 13:**
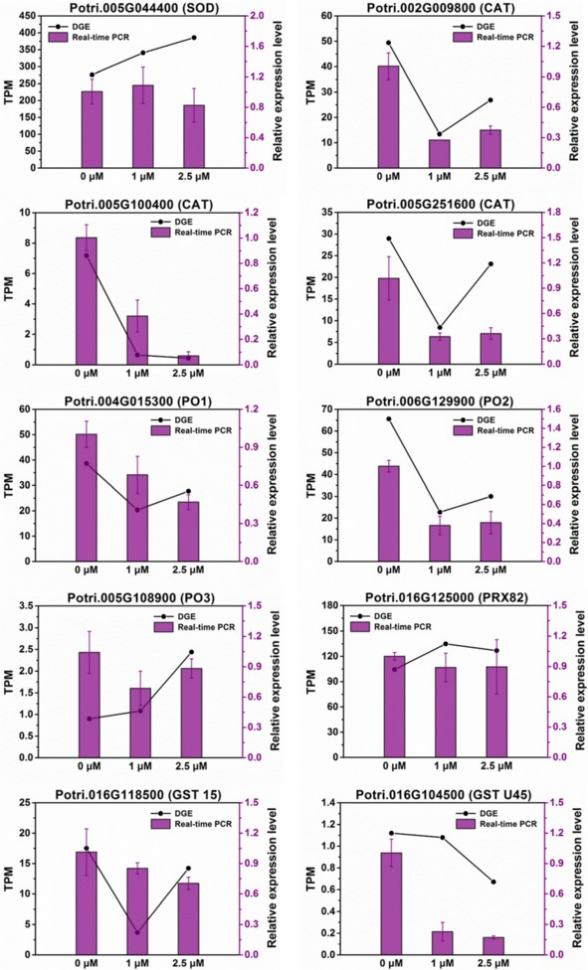
Real-time PCR validations of ROS scavenging genes. Relative level, 2^−△△CT^; TPM, transcript per million mapped reads

In our experiment, the growth of populus roots was inhibited after long-term growth on medium supplemented with TSA (Fig. 2). It is well known that stresses such as salt, cold, drought and heavy metals were able to induce the accumulation of reactive oxygen species (ROS) and inhibit root growth. We examined the ROS accumulation in roots on exposure to different concentrations of TSA for 2 weeks. No significant increases of ROS were observed in roots in response to TSA treatment (Fig. 12). Additionally, we examined the expression levels of genes encoding ROS scavenging enzymes such as superoxide dismutase (SOD), catalase (CAT), peroxidase (POD) and glutathione S-transferase (GTS) in the three DGE libraries and their expression levels were proved by using real-time PCR. A total of 10 genes encoding the four ROS scavenging enzymes were available by searching national center for biotechnology information (NCBI) and they could be detected in the T1 and T2.5 DGE libraries (Additional file 5). Of the ten genes, no one was significantly up-regulated. However, four genes encoding CATs (Potri.002G009800 and Potri.005G100400), PO2 and GST U45, respectively were significantly down-regulated (Fig. 13). Whereas, the expression of the four genes appeared not to be dose-dependent since their expression levels were not significantly different under 1 and 2.5 μM TSA treatments. Base on the analysis of ROS accumulation and the expression of ROS scavenging genes in the roots, we proposed that the inhibition of root growth on exposure to TSA was not due to ROS accumulation.

In our study, the common GO terms for T1 and T2.5 libraries included “active transporter activity”, “response to stimulus” and “response to stress”, implying a relationship between HDACs and stimuli/stress responses in populus roots. Current available data from herbaceous plants such as Arabidopsis, rice and maize have shown that HDACs are involved in stress responses. In plants, the expression of HDAC genes was regulated by abscisic acid (ABA), jasmonic acid (JA), salicylic acid (SA), ethylene, biotic (salt, drought and cold) or abiotic stresses [29–34]. Meanwhile, the alteration of HDAC levels because of overexpression, mutation or RNAi-mediated repression could affect the expression levels of some stress-responsive genes [4]. In our experiment, HDAC activity was inhibited by TSA and some genes encoding ROS scavenging enzymes such as CAT, POD and GST were proved to be down-regulated, suggesting that HDACs were required for ROS scavenging. Based on the evidence from other plants and our current findings, we hypothesized that the inhibition of HDAC activity by TSA might alleviate the control of HDACs on target genes and, as a result, the transcription of some genes involved in stress/stimulus responses were altered. Our data suggested the possibility that stimulus- and stress-responsive genes were directly or indirectly regulated by HDACs in populus roots.

### Root development genes

Root development is a complex process for many plants. Studies of root system in the model plant Arabidopsis advanced the understanding of root development. To date, functions of genes involved in root development, gene regulatory network, mechanisms regulating root development, and root development under stress conditions have been well characterized in Arabidopsis [35, 36]. In Arabidopsis, many genes involved in root development had been identified and they participated in different processes of root development, including patterning and maintenance of the stem cell niche, meristem size control, xylem patterning, root hair pattering, lateral root initiation and patterning, lateral root emergence and auxin pathway [35, 36]. We checked the expression of root development genes in the DGE libraries. A total of 12 genes in T1 and T2.5 libraries were available to be annotated to corresponding genes in Arabidopsis (Additional file 6). During root embryogenesis, root apical meristem (RAM) is established and provides new cells for root formation and growth. At the tip of the RAM, a single layer of initial cells (stem cells) surrounding the quiescent center (QC), a group of less mitotically active cells, form the stem cell niche [35, 36]. Stem cells produce the vascular, endodermal, cortex, epidermal, lateral root cap cells, and columella root cap. QC has a role to maintain the identity of surrounding stem cells by the expression of *wuschel-related homeobox 5* (*WOX5*), which is controlled by clavata3/embryo surrounding region (CLE) peptide CLE40 and the receptor-like kinase Arabidopsis crinkly 4 (ACR4) [35, 36]. The QC identity is specified by plethora (PLT) pathways and short root (SHR)/scarecrow (SCR), transcription factors belonged to the GRAS [gibberellin insensitive (GAI), repressor of ga1–3 (RGA), SCR] family. In Arabidopsis, ACR4 acted as a key factor in promoting formative cell divisions in the pericycle [37] and SHR mutation (*shr*) highly reduced root growth [38]. Vascular system of the plants is consisted of two types of tissues, xylem and phloem, to transport water, nutrients and photosynthates to and from the shoot. Arabidopsis ATHB-8, a member of a small homeodomain-leucine zipper family, is expressed in the vascular tissue and regulates cell proliferation and differentiation. Over-expression of *ATHB-8* in transgenic Arabidopsis reduced the number of lateral roots and higher order roots [39]. Meanwhile, the diameter of the transgenic root was much larger than that of wild-type, suggesting the role of ATHB-8 in secondary growth of root. In populus, in our study, the expression levels of *ACR4* and *SHR* in roots were down-regulated and *ATHB-8* was up-regulated by TSA (Additional file 6). Based on the expression change and corresponding morphological alteration in root development in Arabidopsis, the expression alterations of the genes were consistent with the developmental inhibition and morphological change of root system.

In the differentiation zone of root, one of the key features is the development of root hairs. Root hairs are important for water and nutrient uptake and soil anchoring. Epidermal cells produced in the RAM may become hair cells or nonhair cells based on their relative positions to cells in the underlying cortical layer of the roots. An epidermal cell lies between underlying cortical cells (outside an anticlinal cortical cell wall) will develop as a root hair cell, while an epidermal cell adjacent to a single cortical cell (outside a periclinal cortical cell wall) will develop as a nonhair cell [35, 36]. In Arabidopsis, the cellular pattern of root was determined by six patterning genes, *caprice* (*CPC*)*, enhancer of try and cpc* (*ETC.*)*, glabra 2* (*GL2*)*, GL3*, *enhancer of glabra 3* (*EGL3*)*,* and *transparent testa glabra* (*TTG*). In nonhair cell, a complex of transcription factors GL3, TTG1, EGL3 and WER directly activate transcription of the hair cell fate repressor GL2 and CPC. CPC moves into neighboring cells and inactivates the complex by replacing WER, resulting in the inactivation of GL2 and hair cell specification. Xu et al. (2005) reported that TSA treatment significantly altered the expression of genes *CPC*, *GL2* and *WEREWOLF* (*WER*) in Arabidopsis [7]. In our work, the patterning genes *TTG1* [40–42] and *GL2* [40, 43, 44] were significantly down-regulated after 1 μM and 2.5 μM TSA treatment, respectively. These results suggested the involvement of HDACs in the regulation of root hair patterning in populus.

Root system includes primary roots and LRs. LRs were initiated from pericycle cells adjacent to the xylem poles in differentiation zone. Pericycle cells initiate a series of asymmetric transverse and periclinal divisions and, as a result, a dome-shaped lateral root primordium (LRP) is formed, which leads to the emergence of lateral root [45]. LR development is regulated by auxin, involved complex regulation of auxin biosynthesis, auxin transportation and cellular appropriate response to auxin. The transport of auxin is important for LR development. Arabidopsis AUX1 is a putative auxin influx carrier. Mutation of *AUX1* resulted in a reduction of IAA in root and the mutant (*aux1*) had a reduced number of LRP and fewer lateral roots than wild type [45]. In LR formation and development, many genes are regulated by auxin. The expression of auxin-responsive genes is regulated by two families of important proteins, auxin-response factors (ARFs) and auxin/indole-3-aceticacids (Aux/IAAs). ARFs are transcriptional activator of auxin-responsive genes and positively regulate LR formation, while Aux/IAAs can inhibit the activity of specific ARFs. In presence of auxin, auxin binds to its receptor transport inhibitor response1 (TIR1), which promotes the degradation of Aux/IAA proteins by ubiquitin-ligase complex. The degradation of Aux/IAA proteins derepresses the activity of ARFs, such as ARF7 and ARF19, and allow auxin-responsive genes to be expressed, which leads to the initiation of LR formation [35]. In Arabidopsis, *ARF7* and *ARF19* double mutation (*arf7arf19*) strongly inhibited the lateral root formation at the very early stage of LR initiation [46]. In addition, monopteros (MP)/ARF5 is another important regulator in LR development. The *ARF5* mutant (*arf5-1*) failed to form root meristem [9]. In transgenic Arabidopsis over-expressing *ARF5*, closely positioned lateral root initiation sites and aberrantly spaced lateral root primordia were occasionally observed [47]. These finding indicated that ARF5 is involved in LR formation. In our study, the LR formation and root growth were observed to be inhibited by TSA treatment, especially under 2.5 μM TSA treatment (Fig. 2a). After examination of DEG profiles, several genes related to LR development such as *ARF5* [11], *ARF7* [46] and *auxin resistant 1* (*AUX1*) [45, 48] were found to be differentially down-regulated, even though the fold-changes of the genes were not statistically significant (Additional file 6). Based on functions of the corresponding genes in Arabidopsis, the down-regulation of these genes in populus might contribute, at least in part, to the inhibition of lateral root formation. Additionally, in Arabidopsis, *S-Phase Kinase-Associated Protein 2B* (*SKP2B*), encoding an F-box protein, has recently been reported to play a negatively regulatory role in cell cycle and LR formation [49]. In this study, the promoter of *SKP2B* was regulated by H3 acetylation in an auxin- and IAA14-dependent manner and *skp2b* mutant has longer roots and more LRs than control plants [49]. In our experiment, the expression of *skp2b*-like gene (Potri.005G185700) was not detectable in the roots of plants without TSA treatment, while its expression levels in the roots treated by different concentrations of TSA were much higher, especially under 2.5 μM TSA treatment (Additional file 6). The induction of *SKP2B* might be related to the short root and less LR observed in populus roots after TSA treatments (Fig. 2).

### GA signaling pathway

GAs, as phytohormones, played an essential role in both primary root elongation and LR development [50–52]. Available evidence showed that root growth of many plants was altered due to the change of GA levels such as application of GAs to root system, supplementation of GA-biosynthesis inhibitors, alteration of genes in GA pathway, or mutations of genes involved in GA biosynthesis. GAs may play a negative or positive role in promoting root growth. The work of Gou and colleagues (2010) showed that shortage of GAs promoted root growth and LR development [52]. GA-deficient (35S: *PcGA2ox1*) and GA-insensitive (35S: *rgl1*) transgenic populus exhibited increased LR proliferation and elongation, and these effects were reversed by exogenous GA treatment [52]. Although GAs appeared to have a negative role in root growth and development in Gou and colleagues’ work, GAs played a positive role in promoting root system development in many plants. DELLA proteins function as growth repressors to repress GA signaling. In Arabidopsis, application of GAs to root system promoted root growth by targeting the degradation of DELLA proteins, repressors GA1–3 (RGA) and gibberellin insensitive (GAI), in elongation zone tissues such as epidermal, cortical, endodermal and stele tissues [53]. *GAI* mutation, *gai*, rendered the GAI protein resistance to GA-dependent disruption and the root growth of *gai* mutants was significantly reduced. Uniconazole P (Un-P) is a GA biosynthesis inhibitor. Un-P at the concentrations of 10 and 100 nM were able to significantly inhibit the growth of roots in *Lemna minor* [50]. In pea, the genes *na*, *lh-2*, and *ls-1* encode enzymes catalyzing GA biosynthesis [54]. Mutation of the genes, *na*, *lh-2*, and *ls-1*, reduced GA levels in roots and the length of roots was 50 %, < 15 % and < 15 % decreased, respectively [51]. These findings indicated that GAs played a positive role in promoting root growth. In Arabidopsis, GA biosynthesis was majorly catalyzed by enzymes such as copalyl diphosphate synthase (CPS), ent-kaurene synthase (KS), KO, KAO, GA 20-oxidase, GA 3-oxidase and GA 2-oxidase [52]. In our work, GA biosynthesis pathway in the populus roots subjected to 2.5 μM TSA was significantly altered (Fig. 9). Four genes involved in GA biosynthesis, including *KO*, *KAO*, *GA20ox* and *GA3ox*, were detected to be down-regulated in the roots (Fig. 10). We speculate that the repression of genes involved in GA biosynthesis might be associated with the inhibition of root growth by TSA. Our findings suggested the regulatory role of HDACs in GA biosynthesis.

## Conclusions

Regeneration of roots from shoots and root growth were inhibited by TSA in populus. A digital gene expression (DGE) approach was used to identify differentially expressed genes in the populus roots exposed to different concentrations of TSA. In comparison with the control sample, a total of 1404 and 563 genes were detected to be differentially expressed in the roots subjected to 1 μM and 2.5 μM TSA, respectively. Most of the differentially expressed genes were down-regulated on exposure to TSA. GO and pathway analyses showed that the DEGs were related to many kinds of molecular functions and biological processes. The DGE data provides a large set of candidate genes probably regulated by HDACs in root development.

## Methods

### Plant growth and TSA treatment

Seedlings of *Populus trichocarpa* were cultured under the 25 ± 2 °C, 70–80 % relative humidity, and 16 h light/8 h dark condition. Stems of the populus seedlings were cut into segments each with an axillary bud and cultured on WPM medium containing 0.1 mg/L IBA. Three weeks later, the shoots developed from the axillary buds were cut off and transferred onto the WPM medium supplemented with 0, 1 and 2.5 μM TSA (Sigma) for root regeneration. The populus explants including stem segments, shoots, and regenerated seedlings were cultured under the aforementioned condition. After 2 weeks of rooting, number of roots and root length of regenerated seedlings were determined. Data were statistically analyzed using one-way analysis of variance (one-way ANOVA) followed by Tukey’s multiple comparisons test with significance level set as 5 %. The experiment was repeated three times. The roots grown on medium containing different concentrations of TSA were frozen in liquid nitrogen and kept at −80 °C until RNA isolation.

### HDAC activity assay

Roots grown on the WPM medium supplemented with different concentrations of TSA (0, 1, and 2.5 μM) were collected. HDAC activity in root was measured using the HDAC Colorimetric Assay/Drug Discovery Kit following the manufacturer’s instructions (Enzo Life Sciences). The protein samples were incubated with substrate comprising an acetylated lysine at 37 °C for 30 min. The reaction was stopped by adding developer and incubating the plate at 37 °C for 15 min. The HDAC activity was then measured by microtiter-plate reader at 405 nm. HeLa nuclear extracts were used as the positive control and a blank sample (without enzyme) was used as the negative control. The data were statistically analyzed using one-way ANOVA followed by Tukey’s multiple comparisons test (P level ≤ 5 %).

### Semithin sections

Root sections taken 1 cm from root cap and in the middle region of the roots were fixed immediately in formalin-acetic acid-alcohol (FAA, [55]) for 24 h. After fixation, the samples were dehydrated in ethanol followed by 10 h in 100 % isopropanol and 10 h in 100 % 1-butanol. The dehydrated tissues were then placed in glycol methacrylate (GMA) for infiltration. After this infiltration, the specimens were transferred to GMA and left to polymerize overnight at 60 °C. Sections were stained with toluidine blue and photographs were taken using a microscope (BX43F, Olympus, JP).

### RNA preparation, Solexa/illumine sequencing and data processing

Total RNA was isolated from roots using Trizol reagent (Invitrogen). RNA concentration was determined using a Qubit Fluorometer and RNA integrity was assessed with the Bioanalyzer 2100 (Agilent Technologies). The A260/A280 ratio and A260/A230 ratio of all RNA samples were around 2.1. For Solexa sequencing, the DGE libraries were prepared using Illumina Gene Expression Sample Prep Kit. The single-strand molecules were fixed onto a Solexa sequencing chip (flowcell) and then sequenced by Illumina HiSeq^TM^ 2000 system. In details, mRNA was purified from 6 μg of total RNAs by Oligo (dT) magnetic beads adsorption method. The first and second-strand cDNAs were synthesized using Oligo (dT) primers and digested with restriction enzyme *Nla*III, which recognizes the CATG sites. The 3’ cDNA fragments were purified and the Illumina adaptor 1 was ligated to the 5’ end of the fragments through CATG sticky site. The junction of Illumina adaptor 1 and CATG site is the recognition site of *Mme*I, which is a type of endonuclease with separated recognition sites and digestion sites. It cuts at 17 bp downstream of the CATG site, producing tags with adaptor 1. After removing 3’ fragments with magnetic beads precipitation, Illumina adaptor 2 was ligated to the 3’ ends of the tags, thus generating a tag library with different adaptors at both ends of the tags. After 15 cycles of linear PCR amplification, 105 bp fragments were purified by 6 % TBE PAGE gel electrophoresis. After denaturation, the single-chain molecules were fixed onto the Illumina Sequencing Chip (flowcell). Each molecule turned into a single-molecule cluster sequencing template through *in situ* amplification. Then four types of nucleotides labeled by four different colors were added in, and sequencing was performed with the method of sequencing by synthesis (SBS).

### Data analysis

Raw image data obtained from sequencing was transformed by base calling into sequence data, also called raw data or raw reads. Of the raw data, empty tags (no tag sequence between the adaptors), adaptors, low quality tags (tags containing unknown nucleotides “N”), abnormal tags (too long or too short tags), and single copy tags were removed to obtain clean tags (21 bp). To identity the gene expression patterns in populus roots, all clean tags were annotated by mapping to the sequenced genome of *populus trichocarpa* which covered all possible CATG + 17-nt tag sequences, allowing only 1 bp mismatch. The clean tags mapped to multiple reference sequences were filtered and the remaining clean tags were designated as unambiguous tags. For gene expression analysis, the number of unambiguous clean tags for each gene was calculated and then normalized to transcripts per million clean tags (TPM) [56, 57].

### Analysis and screening of DEGs

Based on the method described by Audic and Claverie [58], a rigorous algorithm was used to identify differentially expressed genes (DEGs) between two samples. The P-value corresponds to differential gene expression test. The false discovery rate (FDR) was used to determine the threshold of P-Value in multiple tests. FDR ≤ 0.001 and the absolute value of | log2Ratio | ≥ 1 were used as the threshold to judge the significance of gene expression difference.

### GO and pathway enrichment analysis

To classify the functions of DEGs, gene ontology (GO) analysis was performed by mapping the DEGs to terms in GO database (http://www.geneontology.org/). For further understanding the functions of the DEGs, pathway enrichment analysis was conducted by searching the KEGG database (http://www.genome.jp/kegg/) [59]. Significantly enriched metabolic pathways or signal transduction pathways in DEGs were identified in comparison to the whole genome background. The calculation formula used for this analysis was as follows:

$$ \mathrm{P}=1-{\displaystyle \sum_{i=0}^{m-1}}\frac{\left(\begin{array}{c}\hfill M\hfill \\ {}\hfill i\hfill \end{array}\right)\left(\frac{N-M}{n-i}\right)}{\left(\begin{array}{c}\hfill N\hfill \\ {}\hfill n\hfill \end{array}\right)} $$

Here N is the number of all genes with a KEGG annotation, n is the number of DEGs in N, M is the number of all genes annotated to specific pathways, and m is the number of DEGs in M. For GO and pathway enrichment analyses, a P-value of 0.05 was selected as the threshold for considering a gene set as significantly enriched.

### Quantitative real-time PCR analysis

The quantitative real-time PCR was set up using SYBR Premix Ex Taq II Kit (TaKaRa) in a volume of 20 μl. The reactions were performed in triplicate for each run and three biological replicates were included. The conditions for the PCR reactions were as follows: 95 °C for 3 m, followed by 44 amplification cycles at 95 °C for 30 s, 55 °C for 30 s, and 72 °C for 30 s. The specific primers used for the selected genes were listed in Additional file 7. For each gene, the pair of primers was designed on different exons using online program Primer 3 (http://bioinfo.ut.ee/primer3/). The Ct values obtained for all the genes were normalized to that of the internal control 18S RNA. For the gene expression analysis, the transcript amount of these genes was determined using 2^-ΔΔCt^ calculations. The transcript level of each gene without TSA treatment (0 μM) was indicated as 1. Transcript levels (n-fold) of the examined genes under TSA treatment conditions were obtained by comparison with their transcript levels in the control sample (0 μM).

### Diaminobenzidine (DAB) stain for hydrogen peroxide

The populus roots were immersed in DAB solution (1 mg/mL, pH 3.8) for overnight. Then the samples were de-stained by soaking in 95 % ethanol and boiling for 10 min.

### Availability of supporting data

The sequence data associated with this study has been deposited to the NCBI Sequence Read Archive (SRA) under the BioProject ID PRJNA304268. Additional supporting data are included as additional files.

## Additional files

Additional file 1:
**KEGG map for nitrogen metabolism.** (TIF 508 kb) Additional file 2:
**KEGG map for ribosome.** (TIF 378 kb) Additional file 3:
**KEGG map for fructose and mannose metabolism.** (TIF 246 kb) Additional file 4:
**KEGG map for glutathione metabolism.** (TIF 244 kb) Additional file 5:
**Expression of genes encoding ROS scavenging enzymes in T1 and T2.5 libraries.** (DOC 41 kb) Additional file 6:
**Expression of annotated genes involved in root development.** (DOC 43 kb) Additional file 7:
**Primers used in real-time PCR to validate genes detected in DGE profiles.** (DOC 36 kb)
