# Supplementary material for: Identification of genes regulated by histone acetylation during root development in Populus trichocarpa
Source: BMC Genomics. 2016 Feb 4;17:96. doi: 10.1186/s12864-016-2407-x (PMC4743431; doi:10.1186/s12864-016-2407-x)
Supplement: Additional file 5: — Expression of genes encoding ROS scavenging enzymes in T1 and T2.5 libraries. (DOC 41 kb) [file 12864_2016_2407_MOESM5_ESM.doc]

Additional file 5 Expression of genes encoding ROS scavenging enzymes in T0, T1 and T2.5 libraries.

| **Gene name** | **Gene ID (phytozome)** | **NCBI accession number** | **Functional annotation^a^** | **TPM** | | |
| --- | --- | --- | --- | --- | --- | --- |
|  |  |  |  | **TSA 0** **µM** | **TSA 1 µM** | **TSA 2.5 µM** |
| SOD | Potri.005G044400 | EF405967 | Cu-Zn superoxide dismutase (SOD) | 276.39 | 340.84 | 385.62 |
| CAT | Potri.002G009800 | XM_002301884 | catalase family protein | 49.4 | 13.42^b^ | 26.86 |
|  | Potri.005G100400 | XM_006382931 | catalase family protein | 7.18 | 0.65 ^b^ | 0.44 ^b^ |
|  | Potri.005G251600 | XM_002306940 | catalase family protein | 28.96 | 8.44 ^b^ | 23.09 |
| POD | Potri.004G015300 | FJ807473.1 | peroxidase (PO1) | 38.62 | 20.34 | 27.75 |
|  | Potri.006G129900 | FJ807474.1 | peroxidase (PO2) | 65.56 | 22.72 ^b^ | 29.97 ^b^ |
|  | Potri.005G108900 | FJ807475 | peroxidase (PO3) | 0.9 | 1.08 | 2.44 |
|  | Potri.016G125000 | KJ201847 | peroxidase (PRX82) | 104.4 | 134.82 | 126.77 |
| GST | Potri.016G118500 | XM_002323555 | glutathione S-transferase (GST 15) family protein | 17.51 | 3.68 ^b^ | 14.21 |
|  | Potri.016G104500 | GQ377238 | glutathione S-transferase (GSTU45) | 1.12 | 1.08 | 0.67 |

^a^ Annotations are based on gene descriptions at <http://phytozome.jgi.doe.gov/pz/portal.html> and blast in [National Center for Biotechnology Information (NCBI)](http://www.baidu.com/link?url=MI8UYLYvDNLeMyEe6yYuAeuQx-5Fv8ZPHdb71ddcWknoOceN-i2iXeMp1RqxWmNt&wd=&eqid=bc18e9600003dfc400000002562dd05f).

^b^, Expression levels of the genes were significantly changed.
