# Supplementary material for: Identification of genes regulated by histone acetylation during root development in Populus trichocarpa
Source: BMC Genomics. 2016 Feb 4;17:96. doi: 10.1186/s12864-016-2407-x (PMC4743431; doi:10.1186/s12864-016-2407-x)
Supplement: Additional file 6: — Expression of annotated genes involved in root development. (DOC 43 kb) [file 12864_2016_2407_MOESM6_ESM.doc]

Additional file 6 Expression of annotated genes involved in root development.

| **Groups** | **Gene ID (phytozome)** | **Functional annotation^a^** | **TPM** | | |
| --- | --- | --- | --- | --- | --- |
|  |  |  | **TSA 0 µM** | **TSA 1 µM** | **TSA 2.5 µM** |
| Root hair patterning | Potri.012G006100 | Transparent testa glabra 1 (TTG1), | 28.96 | 11.47 ^b^ | 21.53 |
|  | Potri.003G052400 | Glabra 2 (GL2) | 20.66 | 11.04 | 8.66 ^b^ |
|  | Potri.015G022000 | Enhancer of try and cpc 1 (ETC1) | 1.35 | 1.3 | 2 |
| Patterning and maintenance of the stem cell niche | Potri.010G138600 | Arabidopsis crinkly 4 (ACR4) | 14.37 | 11.25 | 7.77 |
|  | Potri.007G063300 | Short-root (SHR) | 6.29 | 3.25 | 4 |
| Meristem size control | Potri.010G001000 | Arabidopsis response regulator 1 (ARR1) | 4.04 | 2.81 | 2.44 |
| Xylem patterning | Potri.006G237500 | Arabidopsis thaliana homeobox 8 (ATHB8) | 13.7 | 16.66 | 22.87 |
| Lateral root initiation and patterning | Potri.005G236700 | Auxin response factor 5 (ARF5) | 1.35 | 0.65 | 1.33 |
|  | Potri.006G138500 | Auxin response factor 7 (ARF7) | 3.59 | 1.08 | 2.89 |
|  | Potri.010G078300 | Solitary-root/ indole-3-acetic acid inducible 14 (SLR/IAA14) | 2.69 | 1.73 | 2.44 |
| Auxin pathway | Potri.005G185700 | S-Phase Kinase-Associated Protein (SKP2B) | ND | 0.87 | 2 |
|  | Potri.016G113600 | Auxin resistant 1 (AUX1) | 2.47 | 0.65 | 1.11 |

^a^ Annotations are based on gene descriptions at <http://phytozome.jgi.doe.gov/pz/portal.html> and blast in [National Center for Biotechnology Information (NCBI)](http://www.baidu.com/link?url=MI8UYLYvDNLeMyEe6yYuAeuQx-5Fv8ZPHdb71ddcWknoOceN-i2iXeMp1RqxWmNt&wd=&eqid=bc18e9600003dfc400000002562dd05f).

^b^, Expression levels of the genes were significantly changed.
