# Supplementary material for: Identification of genes regulated by histone acetylation during root development in Populus trichocarpa
Source: BMC Genomics. 2016 Feb 4;17:96. doi: 10.1186/s12864-016-2407-x (PMC4743431; doi:10.1186/s12864-016-2407-x)
Supplement: Additional file 7: — Primers used in real-time PCR to validate genes detected in DGE profiles. (DOC 36 kb) [file 12864_2016_2407_MOESM7_ESM.doc]

Additional file 7 Primers used in real-time PCR to validate genes detected in DGE profiles

| **Gene ID (phytozome)** | **Functional annotation^a^** | **Primer sequences (5’-3’)** | |
| --- | --- | --- | --- |
|  |  | **Up-stream primers** | **Down-stream primers** |
| Potri.001G113400 | Helix-loop-helix DNA-binding superfamily protein | GGTTCAGTGTTGTTAGCTGCA | GCTTCTGTTGCAGCTCAGAA |
| Potri.015G006000 | ATP-binding cassette transporter | TCTCCGCCAGTTCCTTCTAC | CGAGAGAACCAGCTGCTGT |
| Potri.006G107600 | Armadillo/beta-catenin repeat family protein | TTTGCGCCAAGACGTGTTAA | AGGAATTGCGAAACCCCAAC |
| Potri.006G208800 | Integral membrane family protein | CTGGTCCTTTCCCTTCCAT | TGTTTAGGGTAAGTGCAACAGC |
| Potri.019G093800 | Class IV chitinase | AGACCGGACACTTCTGCTAC | TCCACGGCCAAAGTAGTTCT |
| Potri.006G138500 | Auxin response factor 7 (ARF7) | GTTTCCTTGCCTCCAGTTGG | AAGGAAGGTTGGGGTAGCTG |
| Potri.003G133900 | Tiny root hair 1 (TRH1) | CTTCAATACATGGGGCAGGC | AAAGTTGCCATCACCGTGAC |
| Potri.009G170700 | Histone deacetylase (HDA902) | CCCGGCACAGGAGATATACGTGAC | CTCATCGTCAATTCCATCATCCAAT |
| Potri.001G460000 | Histone deacetylase (HDA 904) | ATTCAACTTGCCCTTGTTGG | CTGGAATCTCATTGGGCAGT |
| Potri.002G129700 | Ent-kaurene oxidase family protein (KO) | GGGGCTACTATGTTCCAGCT | TCAGGCTTCCACTCTTCAGG |
| Potri.014G179100 | Ent-kaurenoic acid oxidase (KAO) | TCATGCGCACCTTCATCAA | CTGCATGCTTCAGGTGTTGT |
| Potri.001G176000 | 2-oxoglutarate (2OG)-Fe (II) oxygenase family protein | TGCAACATCTCTAGACGCAAC | CCATTCGGTCTAGCTCTGCA |
| Potri.001G175800 | 2-oxoglutarate (2OG)-Fe (II) oxygenase family protein | ATACCTCAAAGCCCCACACA | TCCACACCAATCCACTCACC |

### ^a^ Annotations are based on gene descriptions at [**http://phytozome.jgi.doe.gov/pz/portal.html**](http://phytozome.jgi.doe.gov/pz/portal.html) and blast in [National Center for Biotechnology Information (NCBI)](http://www.baidu.com/link?url=MI8UYLYvDNLeMyEe6yYuAeuQx-5Fv8ZPHdb71ddcWknoOceN-i2iXeMp1RqxWmNt&wd=&eqid=bc18e9600003dfc400000002562dd05f).
